# Alterations of blood coagulation in controlled human malaria infection

**DOI:** 10.1186/s12936-015-1079-3

**Published:** 2016-01-07

**Authors:** Julia Riedl, Benjamin Mordmüller, Silvia Koder, Ingrid Pabinger, Peter G. Kremsner, Stephen L. Hoffman, Michael Ramharter, Cihan Ay

**Affiliations:** Clinical Division of Haematology and Haemostaseology, Department of Medicine I, Medical University of Vienna, Waehringer Guertel 18-20, 1090 Vienna, Austria; Institute of Tropical Medicine and German Center for Infection Research, University of Tübingen, Tübingen, Germany; Sanaria Inc., Rockville, MD 20850 USA; Clinical Division of Infectious Diseases and Tropical Medicine, Department of Medicine I, Medical University of Vienna, Vienna, Austria

**Keywords:** Malaria, *Plasmodium falciparum*, Coagulation, Controlled human malaria infection (CHMI), Thrombin generation

## Abstract

**Background:**

Alterations of blood coagulation are thought to be involved in malaria pathogenesis. This study had the aim to investigate changes of blood coagulation under the standardized conditions of controlled human malaria infection.

**Methods:**

In a clinical trial aseptic, purified, cryopreserved *Plasmodium falciparum* sporozoites were intravenously (n = 24) or intradermally (n = 6) injected into 30 healthy volunteers. Twenty-two participants developed parasitaemia. Serial blood samples before and during prepatent period and at parasitaemia, diagnosed by microscopic assessment of thick blood smear, were obtained. Biomarkers of blood coagulation (thrombin generation potential, D-dimer, prothrombin fragment 1 + 2, von Willebrand factor, ADAMTS13 activity and soluble P-selectin) were determined.

**Results:**

At first detection of *P. falciparum* parasitaemia, 72.7 % of volunteers had peak thrombin generation 10 % above their baseline. Overall, peak thrombin generation was 17.7 % higher at parasitaemia compared to baseline [median (25th–75th percentile): 225.4 nM (168.1–295.6) vs. 191.5 nM (138.2–231.9); *p* = *0.026*]. There were no significant changes of other coagulation parameters.

**Conclusions:**

The thrombin generation potential, an in vitro blood coagulation test, which reflects an individual´s global coagulation status, was increased by 17.7 % at very early stages of *P. falciparum* malaria, suggesting a hypercoagulable state may be induced, even when parasite density is low.

**Electronic supplementary material:**

The online version of this article (doi:10.1186/s12936-015-1079-3) contains supplementary material, which is available to authorized users.

## Background

Malaria is the most important parasitic disease of mankind, leading to approximately 600,000 deaths per year [1]. The majority of fatal cases are due to infections with the protozoan parasite *Plasmodium falciparum.* Leading causes of death in patients with malaria infection are severe anaemia and cerebral malaria. Moreover, in a review of imported malaria cases requiring intensive care unit admission it was reported that about 10 % of severe malaria cases develop disseminated intravascular coagulation (DIC), a serious coagulopathy associated with poor survival [2]. Also in malaria patients without clinically evident bleeding or thrombotic complications, alterations of the blood coagulation system, such as decreased levels of plasma antithrombin or elevated levels of plasminogen activator inhibitor (PAI)-1, and thrombocytopaenia are frequently found [3, 4]. Thrombocytopaenia is observed in 60–80 % of malaria cases and presents more frequently and severe in complicated *P. falciparum* malaria [4]. In severe malaria patients, also reduced levels of von Willebrand factor (vWF) cleaving protease, ADAMTS13, and increased vWF levels have been found [5, 6]. Interestingly, it has been discussed that the pathogenesis of severe malaria, especially of cerebral malaria, might be closely linked to alterations of the blood coagulation system [7, 8] and alterations of coagulation markers have been used as prognostic markers [8, 9]. Platelets and the coagulation system have important roles in sequestration of infected red blood cells, causing microvessel thrombosis, especially within the brain vasculature. Recently, the link between the innate immune response and coagulation as part of the host defence mechanism has been termed ‘immunothrombosis’ [10], and this phenomenon might be relevant in malaria.

Interestingly, platelets, the cellular component of the coagulation system, were found to be able to directly kill *P. falciparum* parasites [11]. Further research on the interplay between malaria infection and the coagulation system is, therefore, of great interest. Novel and promising coagulation tests that are currently intensively studied include the thrombin generation assay, which is a global coagulation test that reflects the ability of an individual to generate thrombin, the central molecule of the coagulation cascade. Clinical studies showed that this assay correlates with thrombotic and also with bleeding complications in different patient populations [12, 13]. Another currently intensively studied biomarker is soluble P-selectin (sP-selectin), which is a marker of platelet activation. sP-selectin has been extensively studied in patients with vascular thrombotic diseases and might be a promising biomarker of thrombotic events [14, 15].

Standardized investigations of the effects of malaria on humans are difficult to conduct in malaria patients due to frequent co-infections and widely varying clinical manifestations, time of infection and levels of parasitaemia. One way to standardize such investigations is to deliberately infect healthy malaria volunteers by controlled human malaria infection (CHMI). Trials of CHMI are primarily conducted to assess the efficacy of investigational malaria vaccines and drugs. Until recently, these studies were primarily conducted by exposure of volunteers to the bites of *P. falciparum*-infected mosquitoes. In 2013, the first report of CHMI by intradermal injection of aseptic, purified, cryopreserved *P. falciparum* sporozoites (PfSPZ Challenge) was published [16]. The incubation period was prolonged after intradermal injection, and therefore development of a better model using intravenous (IV) injection of PfSPZ was needed. This was accomplished at the University of Tübingen, Germany [17].

In the present study, patient samples from the first study of CHMI by IV injection of PfSPZ were analysed to investigate effects of early *P. falciparum* infection on the human blood coagulation system under well-controlled conditions.

## Methods

### Study design and participants

The current study was performed with specimens from an open-labelled, randomized study of CHMI, which was conducted at the University of Tübingen, Germany [17]. This study was a dose-finding study for CHMI by IV injection of PfSPZ, and had an intradermal (ID) injection control arm. Detailed methods, inclusion criteria and primary outcomes of the study have been previously described [17]. In short, healthy volunteers between 18 and 45 years of age were randomly assigned to the IV or ID groups. In the IV groups, volunteers were injected with escalating doses (numbers of 50–3200) of aseptic, purified, cryopreserved PfSPZ (PfSPZ Challenge, Sanaria, Rockville, MD, USA). On Day 0, PfSPZ were administered and volunteers were examined on the subsequent day (day 1). Thick blood smears were performed twice daily from day 5 until first microscopically detectable parasitaemia, or until day 21 was reached. On the day of the first positive slide or, if no parasites had been detected by then, at day 21 a curative anti-malarial treatment with artemether–lumefantrine was started. Further follow-up visits were performed at day 28, 84 and 168. The primary aim of the original study was to identify an IV PfSPZ Challenge dose that safely infected 100 % (9/9) volunteers. All volunteers gave written informed consent and understanding of the study and procedures were assessed with a quiz. Ethical approval was received from the ethics committee of the University Clinic and the Medical Faculty of the University of Tübingen, Germany. The study was conducted in concordance with the Declaration of Helsinki and was registered with ClinicalTrials.gov, number NCT01624961.

In the present work, parameters of blood coagulation and fibrinolysis were analysed in blood samples obtained at several time points during the study. In each volunteer one baseline blood sample, which was taken at day 0 (before CHMI with PfSPZ Challenge), was drawn for investigation of blood coagulation parameters. In addition, blood samples were obtained at the time of first microscopically confirmed parasitaemia, and 1–3 days before microscopic detection of peripheral blood parasitaemia. The blood sample obtained closest to the day of microscopically detected blood parasitaemia was used for investigation of blood coagulation parameters.

### Laboratory methods

The following coagulation parameters were measured: thrombin generation potential, D-dimer, prothrombin fragment 1 + 2 (F 1 + 2), von Willebrand Factor (vWF), a disintegrin and metalloproteinase with a thrombospondin type 1 motif, member 13 (ADAMTS13)-Activity and soluble P-selectin (sP-selectin).

The in vitro thrombin generation potential was determined with a commercially available assay kit (Technothrombin TGA kit, Techonoclone, Vienna, Austria) on a fully automated, computer-controlled microplate reader (BioTek, FL × 800) and a specially adapted software (Technothrombin TGA, Vienna, Austria) using the fluorogenic substrate Z-Gly-Gly-Arg-AMC (Bachem, Bubendorf, Switzerland) according to the manufacturer’s instructions and as previously described [18]. The reaction was triggered with the TGA RC low reagent, which contained 71.6 pM recombinant human tissue factor lipidated in 3.2 μmol/L phospholipid micelles [phosphatidylcholine (2.56 μmol/L) and phosphatidylserine (0.64 μmol/L)].

D-dimer levels were measured by a quantitative latex assay (STA-LIAtest D-DI; Diagnostica-Stago, Asnieres, France) on an STA-R analyzer (Diagnostica-Stago).

F 1 + 2 levels were measured by ELISA (Enzygnost F 1 + 2; Dade-Behring, Marburg, Germany) according to manufacturer′s instructions. VWF antigen was measured using the latex-enhanced immunoassay ‘STA LIATest VWF’ (Diagnostica Stago, Asniere sur Seine, France). In this assay agglutination-dependent changes in optical density, which are proportional to the VWF concentration, are fully-automatically detected by a STA-R analyser (Diagnostica Stago). ADAMTS 13-activity was determined by a chromogenic ELISA (Technozym, Technoclone, Vienna, Austria). sP-selectin levels were measured using a human sP-selectin ELISA (R&D Systems, Minneapolis, MN, USA) following the manufacturer’s instructions as described previously [15].

### Statistical analysis

Continuous variables were described with the median and the interquartile range (IQR). Categorical variables were described by absolute numbers and percentages. Baseline values (pre-inoculation of sporozoites) of coagulation parameters were compared to values measured at the time of malaria and at the time point 1–3 days before malaria diagnosis, and between groups of volunteers who developed malaria and those who did not develop malaria using paired Wilcoxon’s rank sum tests. To test for changes of coagulation parameters at several time points Friedman’s test was applied. A two-sided *p* value less than 0.05 was regarded as statistically significant. All statistical analyses were performed using IBM SPSS Statistics (Version 21.0, IBM Corp., Armonk, NY, USA).

## Results

### Study participants

In total, 30 healthy volunteers were included into the study. Table 1 shows characteristics of study participants. Groups of three participants were injected with 50 or 200 PfSPZ IV. Groups of nine participants were injected with 800 or 3200 PfSPZ IV. Six participants received 2500 PfSPZ ID. Twenty-two of the volunteers developed *P. falciparum* parasitaemia between day 10 and day 15 after injection of PfSPZ (median: day 12) and nine patients had symptoms of malaria. A detailed report about PfSPZ Challenge doses and infection probabilities, parasite kinetics, safety and tolerability in Tübingen and Barcelona was published [17]. The current study included only samples from participants in Tübingen, Germany.

**Table 1:** Characteristics of study participants (n = 30)

Characteristic	Value
Median age, years (min–max)	26 (21–43)
Sex, number (%)	
Female	7 (23)
Male	23 (77)
Route and dose of PfSPZ Challenge, number Infected/number injected	
IV 50	1/3
IV 200	1/3
IV 800	7/9
IV 3200	9/9
ID 2500	4/6

### Baseline values of blood coagulation parameters in volunteers who developed *Plasmodium falciparum* parasitaemia and in those who did not

Results of the thrombin generation assay, D-dimer, prothrombin fragment 1 + 2, vWF, ADAMTS 13-Activity and sP-selectin at baseline did not differ between those subjects who later developed parasitaemia (n = 22) and those who did not (n = 8) develop parasitaemia [peak thrombin, nM: 191.5 (25th–75th percentile: 138.2–231.9) vs. 184.8 (150.8–221.6), *p* = *0.872*; D-dimer, μg/ml: 0.17 (0.13–0.21) vs. 0.12 (0.07–0.27), *p* = *0.386*; F 1 + 2, pmol/l: 121 (102–152) vs. 114 (86–141), *p* = *0.534*; vWF, IU/ml: 0.89 (0.63–1.07) vs. 0.90 (0.81–1.04), *p* = *0.696*; ADAMTS 13-Activity,  %: 103.5 (91.4–114.0) vs. 99.0 (85.8–119.8), *p* = *0.730* and sP-selectin, ng/ml: 31.1 (27.3–40.2) vs. 26.5 (21.2–35.3), *p* = *0.219*].

### Changes in blood coagulation parameters during *Plasmodium falciparum* infection

Baseline values of the thrombin generation assay, d-dimer, F 1 + 2, vWF, ADAMTS13-Activity and sP-selectin were compared to values measured at the time of peripheral parasitaemia in 22 volunteers who developed parasitaemia. Median peak thrombin generation, the most important outcome measure of the thrombin generation assay, was significantly higher at the time of peripheral parasitaemia in comparison to baseline: 17 of the 22 infected subjects (72.7 %) had peak thrombin generation 10 % above their baseline. There were no differences in thrombin generation between those subjects who were symptomatic (n = 9) and asymptomatic [n = 13; median (25th–75th percentile): 211.4 nM (173.3–248.6) vs. 229.1 nM (159.3–295.6); *p* = *0.845*]. Overall, peak thrombin generation was 17.7 % higher at the time of first detection of *P. falciparum* parasitaemia, compared to baseline. Lag time was significantly shorter at the time of malaria. There was no significant difference in D-dimer, F1 + 2, ADAMTS 13-Activity or sP-selectin. (Table 2).

**Table 2:** Blood coagulation parameters at baseline and at parasitaemia in 22 volunteers who developed parasitaemia

Parameter	At baseline, Median (IQR)	At peripheral blood parasitaemia, Median (IQR)	*p*
Thrombin generation			
Peak thrombin, nM	191.5 (138.2–231.9)	225.4 (168.1–295.6)	*0.026*
Lag time, min	12 (11–13)	10 (8–12)	*0.001*
Peak time, min	19.8 (18.1–22.6)	18.1 (14.6–20.1)	*0.003*
AUC	2836.1 (2494–3377)	2816.6 (2594–3412)	*0.485*
Velocity Index	24.6 (14–20.1)	32.7 (19.5–54.6)	*0.024*
D-dimer, μg/ml	0.17 (0.13–0.21)	0.15 (0.12–0.22)	*0.062*
Prothrombin fragment 1 + 2, pmol/l	121 (102–152)	130 (95–190)	*0.131*
Von Willebrand factor, IU/ml	0.89 (0.63–1.07)	0.81 (0.7–1.04)	*0.833*
ADAMTS13 activity, %	103.5 (91.4–114.0)	99.9 (89.6–117.8)	*0.987*
Soluble P-selectin, ng/ml	31.1 (27.3–40.2)	33.4 (26.3–42.0)	*0.306*
Platelet count, G/l	246 (216–261)	247 (215–271)	*0.911 *

Paired Wilcoxon’s tests *p* values for differences between baseline and parasitaemia are given

Furthermore, changes of coagulation parameters during the incubation period, i.e. at the time before microscopically detectable parasitaemia, were investigated. No significant changes in coagulation parameters measured 1–3 days (median: 2 days) before microscopically detectable parasitaemia in comparison to baseline values were present (data are not shown but can be provided upon request).

**Fig. 1:**
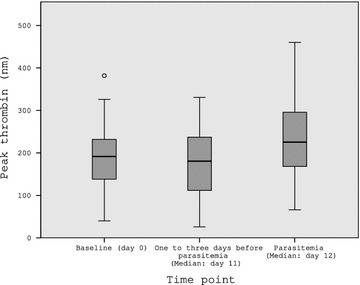
Peak thrombin generation in 22 volunteers. Distribution of peak thrombin generation (nm) at baseline, at the time shortly before microscopically detectable parasitaemia and at the time of parasitaemia is shown. Peak thrombin did not change during the prepatent period, but was significantly higher at the time of parasitaemia in comparison to baseline values. Friedman’s test for differences between time points (*p* = *0.028*)

Figure 1 and Additional file 1: Table S1 show peak thrombin of 22 participants who developed parasitaemia at 3 time points during the study: Baseline values and values measured between 1 and 3 days before parasitaemia were similar, while peak thrombin was significantly higher at the time of microscopically detectable parasitaemia (Friedman’s test for differences between time points *p* = *0.028*).

## Discussion

The present study was done in the context of a project that established a standardized, CHMI protocol by IV administration of PfSPZ, and aimed was to investigate changes in blood coagulation induced by *P. falciparum* infections. The study showed that the coagulation potential of infected volunteers, measured by an in vitro thrombin generation assay, was increased at the time of first detection of parasitaemia, a time at which only 9 of the 22 subjects had symptoms or signs of malaria.

The thrombin generation assay is a global coagulation test that gives information on the potential of an individual to generate thrombin, which reflects the overall haemostatic capacity [12]. Thrombin is the central molecule in the coagulation cascade and measurement of the amount of thrombin that can be generated gives additional information compared to conventional clotting times, such as, for example, the activated partial thromboplastin time (aPTT), which mirrors only the time until the start of clot formation. The thrombin generation assay uses tissue factor (TF) and phospholipids to activate the coagulation cascade and by use of a fluorogenic thrombin substrate a thrombin generation curve can be obtained, which describes the concentration of thrombin generated over time and represents all three phases of coagulation (initiation, propagation and termination) [13]. Several parameters can be read out from the thrombin generation curve, which are the lag phase (time until thrombin burst), peak thrombin (peak amount of thrombin), time to peak, velocity index of thrombin generation curve, and the total amount of thrombin generated [area under the curve (AUC)].

High thrombin generation is associated with hypercoagulable states, such as obesity [18], and increased thrombin generation was shown to predict risk of first and recurrent venous thromboembolism (VTE) [19, 20] and risk of VTE in patients with cancer [21]. Moreover, thrombin generation is diminished in patients with bleeding tendencies and it therefore not only reflects thrombotic, but also bleeding tendency, providing a laboratory test that covers a broader spectrum of haemostasis in comparison to conventional clotting times [13].

The coagulation system is increasingly recognized to play an important role in malaria [7]. Obstruction of small vessels and binding of parasitized red blood cells to endothelial cells are crucial events in the pathogenesis of severe malaria, and endothelial cell activation and activation of the coagulation cascade are proposed to be involved in this process [4]. Several studies have shown altered levels of coagulation parameters in malaria patients [22]. Recent data revealed underlying pathophysiological mechanisms leading to hypercoagulability of malaria patients: Ndonwi et al. showed that the parasite-derived protein, histidine rich protein II (HRPII), has an inhibitory effect on the anti-coagulatory protein antithrombin, which acts physiologically as an inhibitor of thrombin [23]. Furthermore, Moxon et al. found that *P. falciparum*-infected erythrocytes can cause loss of endothelial protein C receptor (EPCR). EPCR physiologically enhances conversion of protein C to activated protein C (APC); APC is an anticoagulatory protein and decreased APC due to loss of EPCR consequently leads to the generation of high levels of thrombin [24]. According to some case reports, recombinant APC (Drotrecogin alfa) was also successfully used for treatment of patients with severe *P. falciparum* malaria who did not respond to standard treatment [25]; however, a clinical trial failed to show benefit for this drug in patients with severe sepsis [26]. Our in vivo data showed that the thrombin generation potential began to change when parasites were at a low density, approximately 5–6 days after release from the liver, and before most subjects were symptomatic. This supports the hypothesis that *P. falciparum* specifically increases thrombin generation potential at the same time as parasites can be first detected by highly sensitive thick blood smear microscopy. However, it also has to be mentioned that in one-third of study participants no change in thrombin generation was observed.

Of note, increase in peak thrombin generation was observed at the first time of microscopically detected parasitaemia in comparison to basic values in our study. Furthermore, lag time, which reflects time until start of clotting, was shorter at first diagnosis of malaria. This observation shows that the thrombin generation assay can capture coagulatory alterations at early stages of *P. falciparum* infection, before other changes in coagulation parameters are observed.

No significant changes in levels of the fibrin degradation products D-dimer and prothrombin fragment 1 + 2 were found in early stage malaria in our study, although these parameters indirectly reflect in vivo thrombin generation. The in vitro thrombin generation potential might be a more sensitive parameter for subclinical coagulation alterations, whereas other parameters might indicate coagulopathy in more advanced or severe malaria cases. In the current study, patients were treated immediately with anti-malarial drugs as soon as first microscopically detectable parasitaemia occurred.

In the current study, also no changes in vWF, ADAMTS13-Activity, sP-selectin or platelet count during early stages of malaria infection were observed. These results are in part contrary to previous studies investigating alterations of coagulatory parameters in *P. falciparum* infection: a study of CHMI induced by bites of *P. falciparum* infected mosquitos found decreased blood platelet counts and elevated levels of vWF at very early stages of malaria infection [27], which was not seen in our study cohort. Underlying explanations for the different results remain obscure. It is unlikely that the differences were related to route of infection (IV injection of PfSPZ versus ID injection of PfSPZ *versus* bites of infected mosquitoes), time point of the assessment during the course of the disease or the severity of the infection. In concordance with previous studies ADAMTS13-activity and sP-selectin remained unchanged in early malaria in this study [27, 28]. However, in symptomatic Indonesian malaria patients ADAMTS13-activity was found to be reduced, indicating that a decrease in this enzyme probably occurs at later stages of malaria [29].

In the current study, alterations of the thrombin generation potential were not observed before microscopic detection of parasites in peripheral blood. Measurement of coagulation parameters in malaria, such as thrombin generation, might therefore not be relevant prior to a microscopically detectable parasitaemia, although the routinely achieved limit of detection of microscopy is usually at least tenfold higher than in this study [17]. Whether the thrombin generation potential might be helpful in predicting severity and outcome of *P. falciparum* infection remains to be studied in appropriate populations, since in the present study parasitaemia was several orders of magnitude lower than in severe or even uncomplicated malaria.

## Conclusions

In conclusion, the thrombin generation potential, an in vitro haemostatic test reflecting an individual´s coagulation status, was significantly elevated at early stages of malaria, reflecting a procoagulant status. The thrombin generation potential shows that the coagulation system reacts early during a *P. falciparum* infection and might have a role in advanced infections and the development of complications.

## Additional file

10.1186/s12936-015-1079-3 Peak thrombin generation at 3 different time points for all 22 individual study subjects who developed parasitemia, including information about PfSZ Challenge route of administration, parasite dose), development of symptoms during infection and parasitemia.
