# Supplementary material for: Alterations of blood coagulation in controlled human malaria infection
Source: Malar J. 2016 Jan 7;15:15. doi: 10.1186/s12936-015-1079-3 (PMC4705755; doi:10.1186/s12936-015-1079-3)
Supplement: Supplementary file 1 — 10.1186/s12936-015-1079-3 Peak thrombin generation at 3 different time points for all 22 individual study subjects who developed parasitemia, including information about PfSZ Challenge route of administration, parasite dose), development of symptoms during infection and parasitemia. [file 12936_2015_1079_MOESM1_ESM.pdf]

Additional Table 1. Peak thrombin generation at 3 different time points for all 22 individual study subjects who developed parasitemia, including information about PfSZ Challenge (route of administration, parasite dose), development of symptoms during infection and parasitemia.

Subject	Route	Dose	Symptoms	Parasites/µl	Peak thrombin, nM		
					At baseline	1-3 days before parasitemia	At parasitemia
1	ID	2500	None	7.5	201.5	237.0	168.1
2	IV	800	None	2.5	255	161.0	295.6
3	IV	200	None	54.0	191.7	152.8	159.3
4	IV	3200	Symptomatic	6.5	241.8	249.9	165.4
5	ID	2500	None	11.0	40.0	84.6	66.2
6	ID	2500	None	8.0	172.9	108.5	119.4
7	IV	800	None	2.5	125.9	111.6	149.7
8	IV	3200	Symptomatic	7.5	325.9	178.7	211.4
9	IV	50	Symptomatic	2.0	92.8	280.9	248.6
10	ID	2500	None	3.5	164.4	328.3	276.9
11	IV	800	None	6.0	231.9	200.2	366.5
12	IV	800	None	6.0	116.3	132.4	229.1
13	IV	800	Symptomatic	9.5	189.3	241.1	233.9
14	IV	800	None	8.5	236.3	236.6	352.9
15	IV	800	None	9.0	199.5	182.1	221.6
16	IV	3200	Symptomatic	5.5	218.9	89.4	460.2
17	IV	3200	Symptomatic	6.5	136.8	134.1	173.3
18	IV	3200	Symptomatic	7.5	138.2	98.6	169.5
19	IV	3200	Symptomatic	10.0	224.7	187.6	207.3
20	IV	3200	Symptomatic	7.0	191.3	236.0	340.6
21	IV	3200	None	9.5	190.6	26.0	241.1
22	IV	3200	None	9	381.8	330.7	431.2
